# Proceedings of the Tennessee Dental Society

**Published:** 1871-12

**Authors:** 


### PROCEEDINGS OF THE TENNESSEE DENTAL SOCIETY.

The fifth annual meeting of the Tennessee Dental Association, was held in Nashville, commencing July 27th 1871, closing on the 29th.

During the two days meeting, peace and harmony prevailed. All present entering into the discussions reported upon by the regular Standing Committees, such as education, physiology, pathology and surgery, histology, therapeutics, operative and mechanical dentistry. Take the meeting altogether, it was one of the best we have had. I am glad to say the society has taken one step toward educating the people in regard to their teeth. At the next meeting there will be a report made upon a circular or pamphlet, the object of which will be to teach the people something of the value of their teeth, as well as the way and means of preserving them. We hope the committee will succeed in getting up something that will be very interesting and instructing to the masses. The next meeting will be held in this place, commencing on the 27th of July. The harmony and interest manifested in this, we take as a sufficient guarantee that the next will be the most interesting we have ever had, each member returned home with a promise to come back next year prepared for the occasion, as well as to do all in their power to interest other good men in the Society.

The election of officers for the ensuing year resulted as follows:

*President*—L. C. Chisholm.
*Vice Presidents*—G. C. Sandusky and J. C. Ross.
*Recording Secretary*—E. S. Chisholm.
*Corresponding Secretary*—A. Hartman.
*Treasurer*—S. J. Cobb.

